# Chitosan Nanocarrier Entrapping Hydrophilic Drugs as Advanced Polymeric System for Dual Pharmaceutical and Cosmeceutical Application: A Comprehensive Analysis Using Box-Behnken Design

**DOI:** 10.3390/polym13050677

**Published:** 2021-02-24

**Authors:** Sara A. Abosabaa, Aliaa N. ElMeshad, Mona G. Arafa

**Affiliations:** 1 Faculty of Pharmacy, Department of Pharmaceutics and Pharmaceutical Technology, The British University in Egypt (BUE), El Sherouk City 11837, Egypt; sara.hakeem@bue.edu.eg; 2 Faculty of Pharmacy, Department of Pharmaceutics and Industrial Pharmacy, Cairo University, Cairo 11562, Egypt; 3 Faculty of Nanotechnology for Postgraduate Studies, Cairo University, El-Sheikh Zayed, Giza 12588, Egypt; 4 Chemotherapeutic Unit, Mansoura University Hospitals, Mansoura University, Mansura 35516, Egypt

**Keywords:** chitosan, caffeine, cellulite, ionic gelation, Box–Behnken Design, optimization

## Abstract

The objective of the present research is to propose chitosan as a nanocarrier for caffeine—a commonly used drug in combating cellulite. Being a hydrophilic drug, caffeine suffers from insufficient topical penetration upon application on the skin. Chitosan nanoparticles loaded with caffeine were prepared via the ionic gelation technique and optimized according to a Box–Behnken design. The effect of (A) chitosan concentration, (B) chitosan solution pH, and (C) chitosan to sodium tripolyphosphate mass ratio on (Y1) entrapment efficiency percent, (Y2) particle size, (Y3) polydispersity index, and (Y4) zeta potential were studied. Subsequently, the desired constraints on responses were applied, and validation of the optimization procedure was confirmed by the parameters exhibited by the optimal formulation. A caffeine entrapment efficiency percent of 17.25 ± 1.48%, a particle size of 173.03 ± 4.32 nm, a polydispersity index of 0.278 ± 0.01, and a surface charge of 41.7 ± 3.0 mV were attained. Microscopical evaluation using transmission electron microscope revealed a typical spherical nature of the nanoparticles arranged in a network with a further confirmation of the formation of particles in the nano range. The results proved the successful implementation of the Box–Behnken design for optimization of chitosan-based nanoparticles in the field of advanced polymeric systems for pharmaceutical and cosmeceutical applications.

## 1. Introduction

Cellulite is a complex metabolic condition that affects more than 85% of post-pubertal females. Its pathophysiology is complex which involves the presence of excess fats in the subcutaneous tissue, resulting in skin irregularities [1]. The skin nodularity caused by the projection of deep fatty layer outward is referred as “orange peel appearance” [2]. Caffeine, a naturally derived alkaloid, is used in multi-diverse industries including food and beverages, health supplements, cosmetology, and pharmaceuticals [3]. It is the prevailing ingredient in topical anti-cellulite products due to its pharmacological activity [4]. It reduces lipogenesis and promotes lipolysis through various mechanisms; principally, it inhibits hormone sensitive lipase (HSL) through inhibition of phosphodiesterase enzymes (PDE) [5]. An increase in the activity of the aforementioned enzymes enhances the degradation of triglycerides. Additionally, caffeine increases the secretion of catecholamine which both activates the β-receptors and blocks the α-receptors in the skin, resulting in enhanced lipolysis and preventing excessive fat accumulation [6,7,8].

The main challenge of tackling cellulite using topical treatments is the presence of lipophilic stratum corneum (SC) which acts as a barrier in preventing the entry of foreign entities including drug molecules [9]. The use of nanotechnology in combating such challenge is expected to enhance drug penetration as reported in previous studies [10,11]. Caffeine has been formulated in different nanosystems such as liposomes [12], ethosomes [13] solid lipid nanoparticles [14], and polymeric nanoparticles fabricated from synthetic polymers such as polycaprolactone [15]. Among the materials used for the production of nanocarriers for caffeine, chitosan (CS) has been also proposed in the literature. Despite the infinite advantages offered by such polymer, the number of studies conducted regarding the incorporation of caffeine in CS is very limited.Accordingly, the research works of Sahudin et al. [16] and Suptijah et al. [17] proposed chitosan as a promising carrier for caffeine. Chitosan possesses numerous benefits including its biodegradability, bioavailability, and high safety profile [18,19]. Most importantly, CS exhibits permeability enhancing properties due to its cationic nature which enhances skin penetration through interacting with the oppositely charged SC, loosening the tight junction, thus enhancing penetration to subcutaneous adipocytes [20,21]. CS nanoparticles are commonly assembled via ionic crosslinking between positive amino groups present in the CS chains with an oppositely charged agent such as sodium tripolyphosphate (TPP)—a multivalent non-toxic anion (TPP) [22,23,24,25]. Due to the aforementioned advantages, it is used as a carrier for a wide array of drugs including herbal extracts [26,27], antimicrobial agents [28], antiviral drugs [29], and anticancer agents [30]. Accordingly, CS–TPP nanoparticles represent the optimum carrier for local drug delivery system intended for topical use.

The Box–Behnken design (BBD), subcategorized from Response Surface Methodology (RSM), is a statistical tool that allows the analysis and evaluation of the main, interaction, and quadratic effects [24]. Moreover, BBD generates optimal formulation according to pre-set desired responses through the optimization of the studied parameters [31]. Construction of formulations is performed through selecting the midpoint lying in the center across each edge in a hypothetical 3D cubic shape. Furthermore, replication of the center point, also referred as “information rich point”, allows estimation of pure experimental uncertainty. This approach of experimental design has proved to be a valuable tool in terms of time saving and economic saving as it allows identification of the interactions by changing the variables simultaneously, thus maximum information can be obtained from a minimum number of experiments [32]. The uniqueness of implementing such design is its inclusion of the midpoints only upon constructing the design and exclusion of the “corner points”, thus avoid any combined factor extremes that might lead to potential loss in the validity of the results [33].

In the present work, caffeine loaded CS–TPP nanoparticles were developed using ionic gelation method to allow an efficient dermal delivery of caffeine to subcutaneous adipocytes at adequate concentration. BBD was utilized for the optimization and investigation of the effects of different factors on desired responses. Encapsulation efficiency percent (EE%), particle size (PS), polydispersity index (PDI), and zeta potential (ZP) were determined for the generated formulations, as well as for the optimal formulation. In addition, microscopic analysis using transmission electron microscope of the optimal formulation was conducted to confirm morphology and size of the prepared nanoparticles.

## 2. Materials and Methods

### 2.1. Materials

Caffeine was purchased from Thermofisher (Kandel, Germany). Low molecular weight CS and TPP were purchased from Sigma-Aldrich Chemical Co. (St. Louis, MO, USA). Acetic acid was purchased from Al-Nasr Pharmaceutical Company (Cairo, Egypt). Sodium hydroxide was purchased from Fisher Scientific (Loughborough, UK). Hydrochloric acid was purchased from Al Ahram Laboratory Chemicals Co. (Cairo, Egypt). Ultrapure water was used throughout this study.

### 2.2. Methods

#### 2.2.1. Experiment Design

The Box–Behnken design, a three-factor and three-level design, was used to statistically optimize the parameters under investigation and to assess the main, interaction, and quadratic effects of the formulation parameters. The design and analysis of 15 experimental runs were developed using Stat-Ease’s Design-Expert-7^®^ (Version 7.0.0, Minneapolis, MN, USA). The factors (independent variables) employed were (A) CS concentration, (B) pH of CS solution, and (C) CS: TPP mass ratio. Low, medium, and high levels of each individual factor presented as −1, 0, and +1, respectively, with their actual values are shown in Table 1. The dependent variables (responses) chosen in the study included EE% [Y1], PS [Y2], PDI [Y3], and ZP [Y4]. The optimum caffeine-loaded CS–TPP nanoparticles formulation was characterized for the previously mentioned responses.

The independent variables range was selected according to previously published literature [22,23,34,35]. Three distinctive zones: clear solution, opalescent suspension, and aggregates, were observed when TPP was added to different concentration of CS. Opalescent suspension indicated presence of very small particles, achieved at a final CS concentration between 0.1 and 0.3%, and TPP concentration between 0.02 and 0.1% [22]. The effect of different pH values (from pH of 3 up to 6) of CS solution on PS, PDI, and ZP have been previously studied [23,34,35]. For this reason, the effect of pH within the pre-selected range was used in the current model. The presence of CS and TPP in specific ratios is considered a critical factor in controlling PS and stability of CS nanoparticles. Therefore, a wide range of CS: TPP mass ratio, varying from 2:1 to 6:1, was studied in the present research.

#### 2.2.2. Fitting of Responses to Optimum Model

The responses attained from each formulation were fitted in different models; namely, linear, 2-factor interaction (2 FI), and a quadratic model. The best fitted model for each response was determined and selected based on the analysis of variance (ANOVA) employing highest R^2^, predicted and adjusted R^2^, and adequate precision [36]. Predicted R^2^ measures the ability of the model to predict a response, while adjusted R^2^ determines the efficacy of variables in improving the model fitting, taking in account the number of variables, thus the presence of insignificant variables tends to decrease the adjusted R^2^ values [32]. Closeness of the predicted and adjusted R^2^, with an approximate difference of 0.2, should be attained to be in a “reasonable agreement” [37]. Adequate precision is also one of the important parameters in selecting the optimum response for a given variable, and is calculated as a signal-to-noise ratio, which should reach a value of >4 to be desired [38]. The selected model was also evaluated for the lack of fit test, and the insignificance of such value relative to pure error indicated the existence of a significant correlation between the chosen independent variables and their responses [39]. The reproducibility of a model is determined through coefficient of variance percent (CV%), which is a percent ratio of the standard error to the mean value of a response, thus a model with a CV% of <10% indicated reproducibility [40].

The composition of fifteen experimental trials with three repetitions of the center point generated by the BBD are illustrated in Table 2. Selection of the most fitted model to each parameter and its significance was performed using analysis of variance (ANOVA), expressed as *p*-value < 0.05. The coefficient of determination (R^2^) was employed for the selection of the best fit model.

#### 2.2.3. Preparation of Caffeine-Loaded CS–TPP Nanoparticles by Ionic Gelation Technique

The ionic gelation method was adopted in the preparation of the CS–TPP nanoparticles [41]. In brief, different concentrations of CS solution in 1% (*v*/*v*) acetic acid were prepared and stirred overnight. The final pH of CS solution was adjusted according to the stated values mentioned in Table 2 using 1 M sodium hydroxide solution for the high pH values and 1 M hydrochloric acid for low pH values. On the other hand, TPP was dissolved in 2 mL of deionised water containing 10 mg caffeine to achieve different CS: TPP mass ratios [27]. The crosslinker solution was sonicated using a water bath sonicator (Elma-Hans Schmidauer: El masonic S60 H, Singen, Germany) for the dissolution of TPP and caffeine. The addition of TPP/caffeine solution into 8 mL CS was performed drop-wisely using a disposable syringe under a magnetic stirrer (Labnet, Accuplate PC 4200, Mexico) until a translucent nanoparticle suspension was formed. The suspension was then stirred for 1 h at 1000 rpm at room temperature to allow complete interaction. The resulting nanoparticles were collected by cooling centrifugation (Centurion Ltd. PRO-Research K241R, Chichester, UK) at 15,000 rpm for 45 min at 4 °C and were washed with deionized water and re-centrifuged at the same conditions to remove excess unreacted soluble CS present in the supernatant. Moreover, the supernatant was used at the end of the experiment to determine drug entrapment efficiency (EE%) [42].

#### 2.2.4. Characterization of Caffeine-Loaded CS–TPP Nanoparticles

##### Entrapment Efficiency Percent (EE%)

The amount of caffeine entrapped within the nanoparticles was determined by indirect method, through calculating the amount of unentrapped drug. The nanosuspension was centrifuged as mentioned above, and the clear supernatant containing the free unentrapped drug was collected, diluted with distilled water and measured spectrophotometrically (Jasco, V-630, Japan) at the wavelength with the maximum caffeine absorbance (λ_max_) 273 nm [43]. Absorbance was converted to the corresponding concentration via a pre-constructed calibration curve [15]. Any interferences that may occur due to unfiltered suspended particles were excluded via spectrophotometric measurement of the supernatant of the unloaded CS–TPP nanoparticles, used as a blank sample [44]. Each sample was measured three times and the mean and standard deviation (SD) were calculated. EE% for each formulation was calculated using the following Equation (1):

(1) $$\mathrm{EE}\%\;=\;\frac{\mathrm{Total}\;\mathrm{Caffeine}\;\mathrm{added}-\mathrm{free}\;\mathrm{Caffeine}\;\mathrm{in}\;\mathrm{supernatan}t}{\mathrm{Total}\;\mathrm{Caffeine}\;\mathrm{added}}\;\times \;100$$

##### Particle Size (PS) and Polydispersity Index (PDI)

Measurements of the PS and PDI were performed on freshly prepared samples, diluted in deionised water and measured at 25 °C using a Malvern Zetasizer (Malvern Instruments Ltd., Malvern, UK). Sample measurements were conducted in triplicate for each preparation and the results were reported in terms of mean diameter (Z-average) ± SD.

##### Zeta Potential (ZP)

Zeta potential (ZP) was measured by a Malvern Zetasizer (Malvern Instruments Ltd., Malvern, UK) by measuring the electrophoretic mobility of the nanoparticles. The stability of nanosuspension is principally governed by the magnitude of its surface charge [45]. The borderline between stable and unstable colloidal system is either positive or negative 30 mV. Accordingly, an absolute value greater than 30 mV is considered stable. [46]. Dilution of freshly prepared samples was performed using deionized water; a portion was injected into a Zetasizer capillary cell attached to electrodes at both ends. Measurements were made at room temperature in triplicate, and results were reported in terms of mean ZP ± SD.

#### 2.2.5. Formulation Optimization

Optimization of the CS–TPP nanoparticles was developed using Design Expert^®^ software (RSM-BBD) after applying specific constraints on the chosen dependent variables. The optimization aimed to maximize EE%, minimize PS and PDI, and maximize ZP. The optimized caffeine-loaded CS–TPP nanoparticles were prepared and measured for the aforementioned responses to ensure the reliability of the developed model.

#### 2.2.6. Characterization of the Optimal-Caffeine Loaded CS–TPP Nanoparticles Formulation

##### Entrapment Efficiency, Particle Size, Polydispersity Index, and Zeta Potential

The EE% PS, PDI, and ZP of optimized CS–TPP nanoparticles was determined as previously described.

##### Transmission Electron Microscope (TEM) Examination

In addition to the previously mentioned characterization techniques, microscopic analysis of the surface morphology and structure of the optimal caffeine-loaded CS–TPP nanoparticles was carried out using TEM (H-600, Hitachi, Tokyo, Japan) [38]. Nanosuspension was diluted using distilled water; a drop was placed over a copper grid coated with carbon film. Imaging of CS–TPP nanoparticles was performed at an operating voltage 

## 3. Results and Discussion

The ability of CS–TPP nanoparticles to entrap hydrophilic drugs such as caffeine along with other parameters was investigated. A design consisting of fifteen formulations and containing three replicated center points, (F5), (F7), and (F11) as demonstrated in Table 2, were developed by BBD and prepared. The results were analyzed and provided considerable information for the optimization of the formulation. Results of all responses including EE% (Y1), PS (Y2), PDI (Y3), and ZP (Y4) are listed in Table 3 as mean ± SD. The data were fitted into different mathematical models and the optimum model was selected based on the optimum R^2^, adjusted R^2^, predicted R^2^, and adequate precision as shown in Table 4.

### 3.1. Effect of Investigated Independent Variables on Entrapment Efficiency Percent (EE%)

Upon fitting the data responses on different models as shown in Table 4, the linear model was the significant model for the EE% analysis with a *p*-value < 0.0001, a *p*-value of 0.8979 for the lack of fit, and the CV% was 6.95%. The regression equation (Equation (2)) of the fitted model for EE% is:

EE% = +14.87 + 8A − 1B − 3.5C(2)

It can be deduced from Equation (2) that the CS concentration (A) had a positive impact on EE%, while the CS solution pH (B) and CS: TPP mass ratio (C) had an inverse relationship with EE%. ANOVA analysis of the final model suggested that A, B, and C factors had a significant effect on the EE% of caffeine, all having a *p*-value of < 0.05. Moreover, the significance of the factors was further confirmed by the high respective F-value of 480.00, 7.50, and 91.88, which indicated that variation between sample means existed.

**Figure 1:**
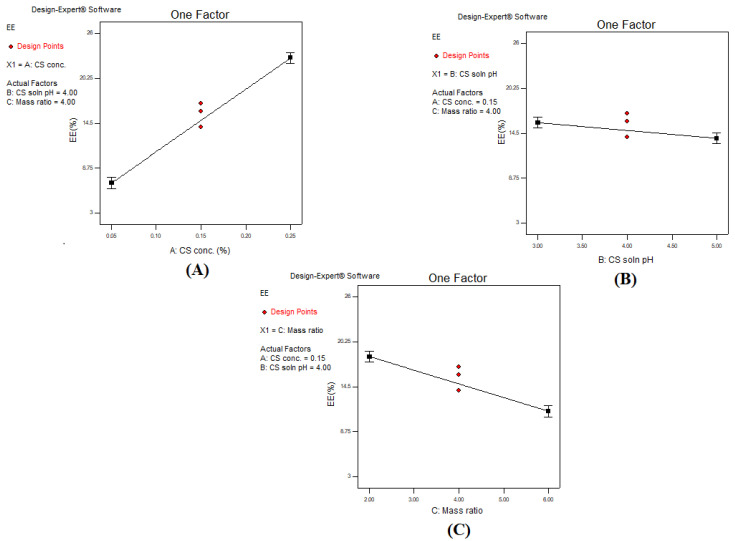
One factor line plot of the main effect of (**A**) chitosan (CS) concentration, (**B**) CS solution pH, and (**C**) chitosan: sodium tripolyphosphate (CS: TPP) mass ratio on EE%.

Entrapment efficiency had a positive linear relationship upon increasing concentration of CS solution as shown in the one factor plot in Figure 1A. A maximum EE% of 26% (F13) was attained at a CS concentration of 0.25%. At a low CS concentration (0.05%), a low EE% value of 10% was observed at a constant CS:TPP mass ratio and pH in comparison to (F13). Increasing the CS concentration increased availability of the protonated CS (–NH_3_^+^) in the system, confirmed by elevation of ZP. Accordingly, availability of the binding sites for the crosslinker increased, hence higher EE% [42]. These results were in agreement with Kalam et al. [47], confirming the decrease in EE% was due to a lower concentration of CS used. Contrarily, increasing the pH of CS solution while maintaining constant values of the other factors resulted in a significant reduction in the EE%; however, it should be noted that the reduction is not intense as shown in Figure 1B. This was demonstrated by (F6) with pH 3, which achieved an EE% of 13.8 ± 0.52%, which was reduced to 11.11 ± 0.24% upon increasing pH to 5 (F8). This could be explained due to the decrease in the protonation of CS molecule at higher pH conditions. Thus, the capacity of the CS to ionically interact with TPP ions was reduced resulting in a lower EE%, despite the larger size of formed nanoparticles [36]. Moreover, the CS: TPP mass ratio had an inverse effect on EE% upon increasing the ratio from 2:1 to 6:1. As observed from Figure 1C, the EE% decreased significantly upon increasing the CS: TPP mass ratio from 2:1 to 6:1. This could be demonstrated by comparing (F13) having an EE% of 26.34 ± 0.52% when the CS: TPP mass ratio is 2:1, which was reduced to 19.25 ± 0.1% upon increasing the mass ratio to 6:1 as in (F15). This could be explained due to the presence of a low amount of TPP anions available for crosslinking with CS. Low EE% was confirmed by the reduced particle size.

The low EE% could be attributed to the hydrophilic nature of caffeine and its small molecular weight which led to its complete loss into the hydrophilic phase, rather than its entrapment in the formed CS–TPP nanoparticles, which is in agreement with Bodmeier et al. [48]. These findings were also supported by Lazaridou et al. [49], who reported that the low EE% of deferoxamine mesylate upon its entrapment in CS–TPP nanoparticles was due to its hydrophilic nature. This confirms that hydrophilic drugs have a low EE% in CS–TPP nanoparticles due to their high water solubility, thus partitioning to the aqueous phase.

Another prospective to be considered in interpreting EE% is the interaction of caffeine with the positively charged CS molecules. Based on caffeine chemical structure, it tends to protonate when it dissolves in water [15,50]. Positively charged caffeine interacted with the positively charged CS present in the acidic media, leading to repulsion between the two moieties due to similar charges. This repulsion is expected to contribute to the low entrapment of caffeine within the chitosan polymeric nanoparticles. In a similar trend, Janes et al. [51] assessed the ability of CS–TPP nanoparticles to entrap the positively charged hydrophilic drug doxorubicin, similar to caffeine. Their results revealed a low EE% reaching 9.1%, which was explained by the presence of repulsion between a similarly charged polymer and drug.

Such results of low entrapment efficiency of caffeine were also attained using other nanocarriers as reported in the literature, including liposomes reaching 10% entrapment [52]. Furthermore, low values of EE% was also witnessed by Ascenso et al. [53] upon incorporation in vesicles including transfersomes, ethosomes, and transethosomes reaching a value of less than 10%, which confirms that the hydrophilicity of caffeine molecules is responsible for its low entrapment within the polymeric network.

### 3.2. Effect of the Investigated Independent Variables on Particle Size (PS)

The fundamental approach in utilizing nanoparticles in topical drug delivery products is to exploit their small size to overcome the SC barrier and facilitate drug penetration [10]. Thus, it is essential to target nanoparticles with low PS for successful delivery of caffeine. The quadratic model was the significant model as shown in Table 4 with a *p*-value of < 0.05; an insignificant lack of fit value was obtained in this model with a *p*-value of 0.4739, and CV% was 8.52. The final equation (Equation (3)) to correlate the three independent variables and PS was as follows:

PS = +197.33 + 232.6A + 66.47B − 143.38C + 54.8AB − 113AC − 29.25 BC + 134.11 A^2^ + 36.86 B^2^ + 45.06C^2^ (3)

**Figure 2:**
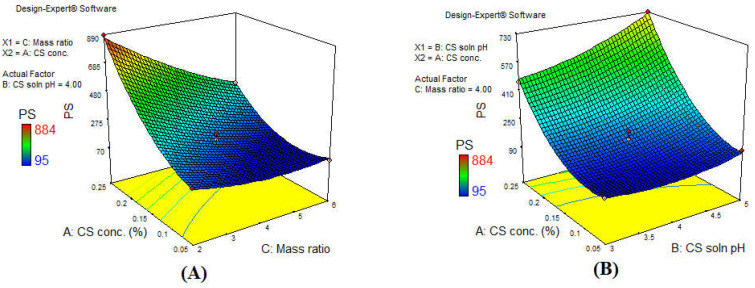
3D surface plot response of the main effect of (**A**) CS concentration and CS: TPP mass ratio, and (**B**) CS concentration and CS solution pH on the PS.

According to the ANOVA analysis, the three independent variables studied had an effect on the PS as all formulations had a *p*-value of < 0.05 and the F-value for each of the factors was 610.49 for (A), 49.86 for (B), and 231.96 for (C). PS of all the prepared formulations ranged from 95 ± 5 to 884 ± 7 nm as presented in Table 3. The 3D surface plot, illustrated in Figure 2A, showed that increasing the concentration of CS along with increasing the TPP amount for the crosslinking (low mass ratio of 2:1) resulted in the largest PS formation. CS is present as extended chains at a low concentration which facilitates dispersion of TPP anions to the exposed positively charged amino groups of CS. This rapid dispersion leads to the formation of small compact nanoparticles. However, upon increasing CS concentration, the molecules become entangled in such a way that it hinders TPP anions dispersion within CS molecules, thus inefficient crosslinking occurs, and hence larger particles are formed [23,54]. Moreover, at a low CS: TPP mass ratio (2:1), the presence of excess TPP anions above equilibrium leads to interaction with the amino groups in CS and the formation of enlarged particles with lower surface charge. This can also be seen by comparing the PS of (F4) (95.33 ± 5.03 nm) with a CS:TPP of 6:1, which increased rapidly on reducing the CS:TPP mass ratio to 2:1 in (F2), reaching a PS of 181.003 ± 8.54 nm while maintaining the same CS solution pH and concentration. Nanoparticle formation principally depends on the formation of inter and intra-molecular interaction between CS chains and multivalent TPP. Thus, at a high CS: TPP mass ratio (small quantity of TPP is available in comparison to CS), TPP crosslink with CS forming small non-aggregated nanoparticles. However, as the CS:TPP mass ratio declines (larger quantity of TPP is available in comparison to CS) as in the current study, CS molecules were fully crosslinked with the presence of excess TPP, which resulted in large aggregated particles and precipitation forming a turbid suspension. This result was in agreement with Bing Hu et al. [55], who suggested that PS decreased linearly with increasing CS: TPP mass ratio. This phenomenon was also witnessed by Papadimitriou et al. [56], Aziz et al. [57], Leelapornpisid et al. [58], and Perinelli et al. [59] who elucidated that at smaller CS: TPP mass ratios, the amount of TPP was in excess, linking the nanoparticles together to form larger particles. This effect was further augmented upon increasing the pH of the CS solution, due to the decreased protonation of CS molecules leading to decreased crosslinking ability with TPP. Along these lines, a pattern of increased PS was witnessed upon increasing the CS solution pH, (F6) having a PS of 121.33 ± 9.07 nm at a pH of 3, which increased significantly to a value of 175.67 ± 5.86 nm in (F8) upon increasing pH to 5. The protonation degree of CS decreased as the pH of the solution increased, leading to reduction in its capacity to crosslink with TPP and the formation of large non-compact particles. [36]. This could be attributed to the change in the conformation of CS molecule from an extended highly protonated form in the acidic medium, to a less protonated folded form in a high pH medium. The folding, referred to as loop conformation of the CS chain, also resulted in fewer amino groups being exposed to the TPP anions, lower crosslinking, and eventually the formation of CS–TPP nanoparticles with larger PS. These findings are in agreement with Abd-Allah et al. [23]. The effect of the CS solution pH and CS concentration on PS at a CS: TPP mass ratio of 4:1 is demonstrated in the 3D surface plot in Figure 2B, showing that the effect of pH on PS had a much higher impact upon the formulation using a higher concentration of CS.

### 3.3. Effect of the Investigated Independent Variables on Polydispersity Index (PDI)

Another important parameter to be considered during formulation is the size uniformity of the nanoparticles, referred to as “polydispersity index” (PDI) [60]. It is a dimensionless numerical value that ranges from 0.0, indicating a highly uniform size distribution, to 1.0, which is attained when samples have non-uniform particle size distribution [61]. Along these lines, the PDI in our current study ranged from 0.24 ± 0.008 (F2) up to 0.753 ± 0.004 (F14), displayed in Table 3, indicating that a number of the prepared formulations had an acceptable PS distribution.

In a similar trend to PS, the quadratic model was the most suitable model in analyzing the effect of the three factors on PDI (Table 4). ANOVA analysis of the final model indicated that all factors, A, B, and C, significantly affected the PDI with a respective F-value of 807.13, 130.78, and 40.82. Insignificant lack of fit value with a *p*-value of 0.7374 and CV% of 4.7 were obtained in this model. The regression equation (Equation (4)) of the quadratic model for the PDI was:

PDI = +0.27 + 0.19A + 0.076B − 0.042C + 0.067AB − 0.051AC + 0.015BC + 0.10A^2^ + 0.062B^2^ + 0.076C^2^ (4)

**Figure 3:**
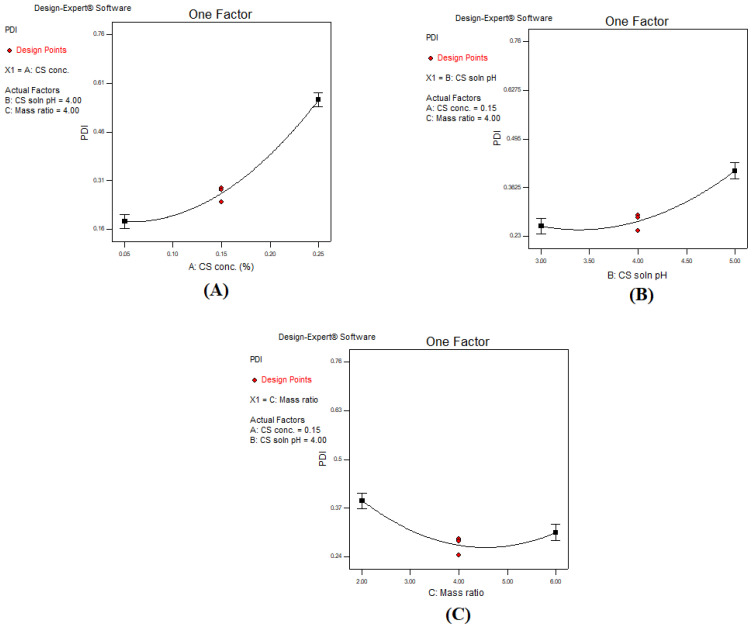
One factor line plot of the main effect of (**A**) CS concentration, (**B**) CS solution pH, and (**C**) CS:TPP mass ratio on the PDI.

**Figure 4:**
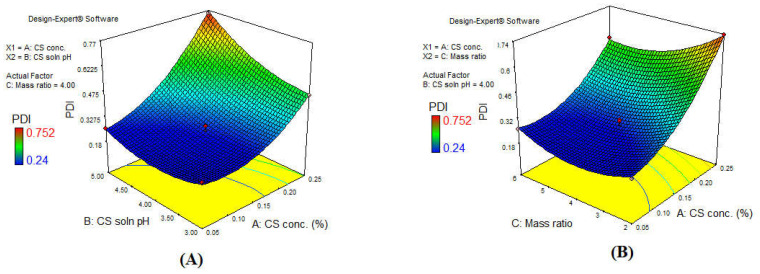
3D surface plot showing the impact of the interaction of (**A**) CS solution pH and CS concentration, and (**B**) CS: TPP mass ratio and CS concentration on the PDI.

The pattern of PS variation in our present study linearly correlated with PS distribution, meaning that an increase in PS was accompanied with an increase in PDI. This was consistent with the data reported by Fan et al. [62]. As previously described in detail in Section 3.3, an increase in CS concentration and CS solution pH resulted in an increase in PS. Increasing CS concentration above a certain level was accompanied by an increase in the electrostatic repulsion between CS molecules, leading to an increase in the intermolecular hydrogen bond formation. Hence, the rearrangement and aggregation of CS–TPP nanoparticles occurred with variable sizes and increased PDI value as seen in Figure 3A [63]. However, increasing the CS solution pH reduced the CS protonation and the crosslinking ability of CS with oppositely charged TPP; thus, the probability of the formation of compact CS–TPP nanoparticles was reduced, and larger heterogeneous particles were formed as seen in Figure 3B [23]. On the contrary, increasing the CS: TPP mass ratio reduced the PDI (Figure 3C). Decreasing the CS: TPP mass ratio resulted in the formation of larger particles when compared to increased mass ratio. This was due to the higher availability of TPP at a low mass ratio, bearing in mind that TPP is a multivalent (penta) anion with a capacity of forming five ionic bonds with amino group present in CS, which led to formation of larger aggregated particles [64]. The intensity of the impact of each of the factors is demonstrated in the 3D surface plot in Figure 4: increasing the CS concentration increased the PDI intensely, while the CS solution pH resulted in moderate increase in the PDI. Alternatively, a decrease in the CS: TPP mass ratio slightly increased PDI, indicating that the impact of CS concentration and pH was greater than that of mass ratio [65].

### 3.4. Effect of the Investigated Independent Variables on Zeta Potential (ZP)

Stability of the colloidal system is determined by measuring the surface charge, referred as zeta potential. The surface charge depends on the type of constituents used in the CS–TPP nanoparticles. The linear model was the most optimum model for the ZP as presented in Table 4, with a *p*-value of < 0.05, an insignificant lack of fit value of 0.4659, and CV% of 8.98% were attained. ANOVA analysis indicated that only the CS solution pH and CS: TPP mass ratio had a major significant effect on the ZP with a *p*-value of < 0.05 and respective F-values of 76.5 and 9.43. The regression equation of the chosen model was as follows (Equation (5)):

ZP = +30.84 + 2.02A − 8.54 B + 3.00 C(5)

As shown from Table 3, all formulations showed a positive value that ranged from a minimum of 22.0 ± 2.00 mV (F10) to a maximum of 44.6 ± 0.51 mV (F6). The positive charge of CS–TPP nanoparticles, arising from the amino groups, is advantageous as it facilitates skin penetration [66,67] since skin surface is negatively charged due to the presence of both phosphatidylcholine [68] and carbohydrates [69] containing negatively charged groups.

**Figure 5:**
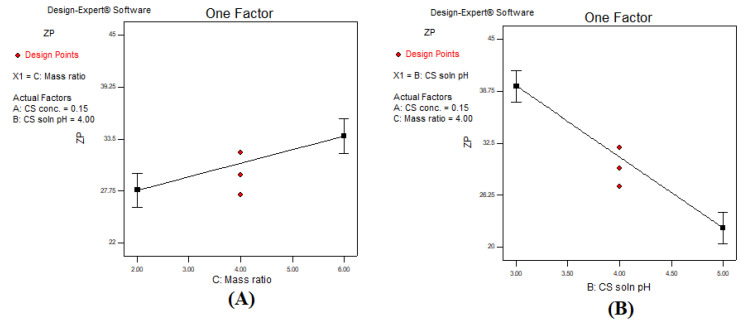
One factor line plot of the main effect of (**A**) CS: TPP mass ratio, and (**B**) CS solution pH on the ZP.

A positive correlation between the CS: TPP mass ratio and ZP is observed in Figure 5A. This was harmonious with Fan et al. [54] who interpreted that the reduction in ZP upon using lower mass ratios was due to neutralization of CS positively charged amino groups by TPP anions. A similar result was stated by Gan et al. [70], Stoica and Ion [71], and Ing et al. [55] who observed an intense decline in the ZP upon reducing the CS to TPP ratio. Moreover, Pooja et al. [72] found similar results and suggested that the increased surface charge upon increasing the mass ratio of CS:TPP was due to the increased availability of free positive amino groups within the CSS–TPP complex. In an opposite manner, increasing the pH of CS solution resulted in a significant reduction in the ZP (Figure 5B). This was due to the deprotonation of the CS molecule amine group which led to a reduction in the net positive charge of the CS–TPP nanoparticles, hence decreased ZP values [44]. Moreover, Rázga et al. [73] observed an increase in ZP value upon reducing the pH from 6.0 to 3.5 (more acidic environment), from a value of 23 ± 1 to 38 ± 3 mV due to the protonation of the CS amine functional groups, with a subsequent conformational rearrangement from the coiled form to an extended form.

### 3.5. Formulation Optimization

After the statistical analysis of the fitted models, optimization of the formulation was conducted using Design Expert^®^. Upon applying the desired constraints, maximum EE% and ZP, and minimum PS and PDI, a formulation with a calculated desirability of 0.805 was selected. The optimized level for each factor for the optimal formulation concluded by the software were as follows: the CS concentration used was 0.19%, a CS solution pH of three, and a CS: TPP mass ratio of 5.26:1. The formulation prepared under the aforementioned values were further characterized to assess the validity of the optimization process.

### 3.6. Characterization of the Optimal Caffeine-Loaded CS–TPP Nanoparticles

#### 3.6.1. Determination entrapment Efficiency (EE%), Particle Size (PS), Polydispersity Index (PDI), and Zeta Potential (ZP)

**Figure 6:**
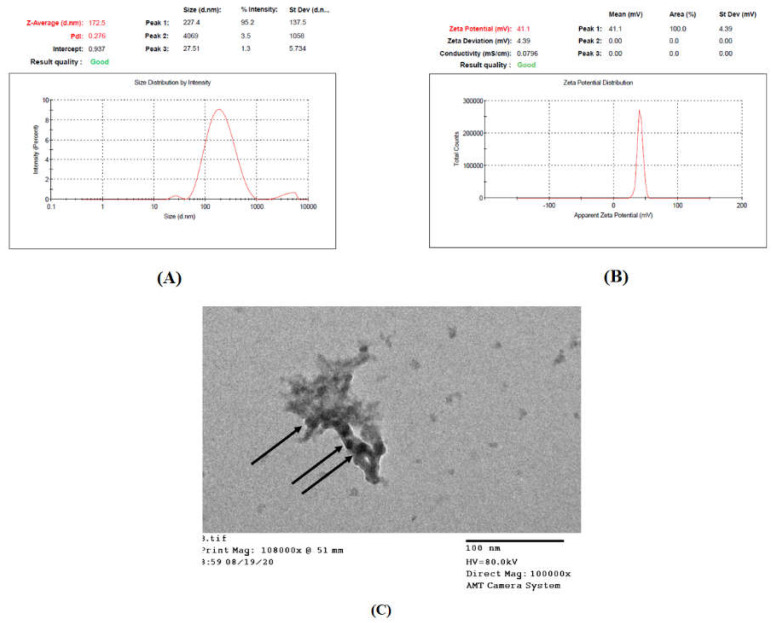
Optimized formulation characterization: (**A**) size distribution report, (**B**) zeta potential report, and (**C**) TEM image of the optimal formulation showing dense particle aggregation within a network.

The observed values of the tested responses, EE%, PS, PDI, and ZP are shown in Table 5, It is important to note that the observed values were in very close agreement with the predicted values obtained by the software which confirm the validity of the optimization process and the high fitting of the chosen model to represent each response tested. Moreover, this was further confirmed as the values observed practically lay between the low and the high confidence interval present in Table 5. The size distribution (intensity-based) and the ZP reports of the optimal formulation are shown in Figure 6, displaying a “Good” quality result of the report indicating that the sample meets the quality criteria.

#### 3.6.2. Transmission Electron Microscope (TEM) Examination

Figure 6C confirmed the spherical nature of caffeine-loaded CS–TPP nanoparticles with an average size of 30 to 40 nm. The appearance of particles aggregated in a network was consistent with the findings of Keawchaoon et al. [74]. The smaller PS displayed by TEM was much smaller than the size detected by dynamic light scattering techniques (Zetasizer device). It must be highlighted that the latter technique depends on determining the hydrodynamic diameter of the particles, hence larger diameters are expected due to the swelling of the CS polymer, as well as the aggregation of particles upon their dispersion in water. In a different manner, TEM measures solely the exact diameter of a single individual particle, therefore an actual smaller size is observed. This was also reported by several other authors [24,75].

## 4. Conclusions

Caffeine-loaded CS–TPP nanoparticles were prepared successfully using the ionic gelation technique. The effect of three independent variables; namely, CS concentration, CS solution pH, and CS: TPP mass ratio on the entrapment efficiency percent, particle size, polydispersity index, and zeta potential, were extensively studied. Statistical analysis and optimization were performed using the Box–Behnken design; an optimal formulation with a desirability of 0.805 was deduced. Despite the satisfactory results of PS 173.03 ± 4.32 nm, PDI 0.278 ± 0.01, and a ZP of 41.7 ± 3.0 mV, the EE% showed a relatively low value of 17.25 ± 1.48%, which was as a result of multiple factors including caffeine hydrophilicity and low molecular weight alongside its positively charged nitrogens. The demonstrated results confirmed the successful utilization of the Box–Behnken design as a tool for analysis and optimization of polymeric nanoparticles fabricated from naturally occurring chitosan to be implemented in the field of advanced polymeric systems for pharmaceutical application.

## Figures and Tables

**Table 1:** Independant responses with their respective levels.

Factors (Independent Variables)	Levels		
	Low (−1)	Medium (0)	High (+1)
(A) CS * conc (%)	0.05	0.15	0.25
(B) CS solution pH	3	4	5
(C) Mass ratio CS:TPP	2:1	4:1	6:1

* CS: Chitosan.

**Table 2:** Composition of generated formulations using the Box–Behnken design (BBD).

	Factor Levels in Their Actual Value		
Formulations	CS Concentration (%)	pH	Mass Ratio (CS:TPP)
F1	0.05	3	4:1
F2	0.05	4	2:1
F3	0.05	5	4:1
F4	0.05	4	6:1
F5	0.15	4	4:1
F6	0.15	3	6:1
F7	0.15	4	4:1
F8	0.15	5	6:1
F9	0.15	3	2:1
F10	0.15	5	2:1
F11	0.15	4	4:1
F12	0.25	3	4:1
F13	0.25	4	2:1
F14	0.25	5	4:1
F15	0.25	4	6:1

**Table 3:** Responses on encapsulation efficiency percent (EE%), particle size (PS), polydispersity index (PDI), and zeta potential (ZP) of the generated formulations.

Formulations	EE ± SD (%) (Y1)	PS ± SD (nm) (Y2)	PDI ± SD (Y3)	ZP ± SD (mV) (Y4)
F1	7.09 ± 1.81	120.2 ± 2.01	0.249 ± 0.004	39.5 ± 4.11
F2	10.13 ± 0.91	181.003 ± 8.54	0.24 ± 0.008	26.0 ± 4.00
F3	5.19 ± 1.03	163.33 ± 7.64	0.261 ± 0.004	23.3 ± 1.15
F4	3.45 ± 0.52	95.33 ± 5.03	0.263 ± 0.004	27.0 ± 1.00
F5	17.22 ± 0.24	182.00 ± 3.00	0.287 ± 0.003	29.5 ± 5.07
F6	13.8 ± 0.52	121.33 ± 9.07	0.273 ± 0.004	44.6 ± 0.51
F7	16.62 ± 0.29	226.00 ± 10.15	0.281 ± 0.004	32.0 ± 2.00
F8	11.11 ± 0.24	175.67 ± 5.86	0.459 ± 0.005	24.7 ± 3.06
F9	19.82 ± 0.59	323.67 ± 7.77	0.392 ± 0.009	34.6 ± 4.13
F10	17.92 ± 0.41	496.00 ± 7.55	0.517 ± 0.031	22.0 ± 2.00
F11	14.06 ± 0.43	184.00 ± 6.00	0.245 ± 0.005	27.3 ± 1.15
F12	23.02 ± 0.24	463.67 ± 12.66	0.473 ± 0.028	43.7 ± 1.53
F13	26.34 ± 0.52	883.67 ± 7.37	0.736 ± 0.016	27.0 ± 3.00
F14	21.15 ± 0.19	725.67 ± 6.11	0.753 ± 0.004	24.0 ± 2.00
F15	19.25 ± 0.1	345.67 ± 8.51	0.553 ± 0.039	37.3 ± 2.52

**Table 4:** ANOVA analysis of the investigated responses.

Model	R^2^	R^2^ Adjusted	R^2^ Predicted	Adequate Precision	Remarks
Entrapment efficiency percent (Y1)					
Linear	0.9814	0.9763	0.9705	43.125	Suggested
2FI	0.9814	0.9674	0.9445	27.801	-
Quadratic	0.9923	0.9793	0.9833	29.158	-
Particle size (Y2)					
Linear	0.8155	0.7652	0.7652	12.766	-
2FI	0.9013	0.8272	0.8272	11.249	-
Quadratic	0.9954	0.9872	0.9872	35.272	Suggested
Polydispersity index (Y3)					
Linear	0.7834	0.7244	0.6492	11.002	-
2FI	0.8499	0.7374	0.6757	8.52	-
Quadratic	0.996	0.9888	0.9684	34.508	Suggested
Zeta Potential (Y4)					
Linear	0.8913	0.8617	0.7871	16.182	Suggested
2FI	0.9405	0.8959	0.7666	14.098	-
Quadratic	0.9768	0.9351	0.8235	14.94	-

**Table 5:** Validation of the optimization process.

Response	Predicted Value	Observed Value	Low Confidence Interval	High Confidence Interval
EE%	16.8412	17.25 ± 1.48	15.68	18.00
PS (nm)	177.267	173.03 ± 4.32	128.43	226.11
PDI	0.303	0.278 ± 0.01	0.27	0.34
ZP (mV)	42.067	41.7 ± 3	38.96	45.17
